# No-Touch Aorta Off-Pump LIMA-Radial Artery Y-Graft CABG as a Safe Strategy for All-Comers: Long-Term Survival

**DOI:** 10.3390/jcm14144878

**Published:** 2025-07-09

**Authors:** Tomasz Plonek, Dominik Mendyka, Frank R. Halfwerk

**Affiliations:** 1 Department of Cardio-Thoracic Surgery, Thorax Centrum Twente, Medisch Spectrum Twente, Koningstraat 1, 7512 KZ Enschede, The Netherlands; 2 Department of Cardiac Surgery, MEDINET Heart Center Ltd., 51-124 Wrocław, Poland; 3 Faculty of Medicine, Wroclaw Medical University, 50-367 Wrocław, Poland; 4 Department of Biomechanical Engineering, TechMed Centre, University of Twente, 7522 NB Enschede, The Netherlands

**Keywords:** coronary artery bypass grafting, off-pump, myocardial revascularization, no-touch, anaortic

## Abstract

**Objectives:** To assess the long-term survival outcomes of patients undergoing no-touch aorta, total arterial off-pump coronary artery bypass grafting (OPCAB) using a left internal mammary artery (LIMA)–radial artery (RA) Y-graft configuration. This approach was applied uniformly to all-comers undergoing isolated CABG between 2004 and 2021, irrespective of preoperative risk profile. **Methods:** We included all patients treated with total arterial OPCAB using the LIMA–RA Y-graft without additional concomitant procedures. Patients were stratified into five age groups (<50, 50–59, 60–69, 70–79, and >80 years). Survival at 5 years was analyzed using Kaplan–Meier curves and Cox regression analysis. **Results:** A total of 2174 patients were analyzed, with a median follow-up of 3266 days. In-hospital mortality was 0.6%, whereas postoperative stroke was 0.3% without residual trauma and 0.2% with residual trauma, respectively, without differences between age groups. The mean number of grafts per patient was 3.7, with no significant variation between age groups (*p* = 0.09). Overall, 5-year survival was 90% (n = 1767), ranging from 98% in the youngest group to 65% in the oldest (log-rank *p* < 0.0001). **Conclusions:** No-touch aorta, total arterial OPCAB using the LIMA–RA Y-graft is a safe and effective revascularization strategy for a broad spectrum of patients, including those with advanced age and comorbidities.

## 1. Introduction

Coronary artery bypass grafting (CABG) can be performed either with cardiopulmonary bypass (on-pump CABG) or without it (off-pump CABG, OPCAB), using various vascular graft configurations. The optimal surgical technique remains a matter of ongoing debate. According to the 2018 ESC/EACTS guidelines on myocardial revascularization, OPCAB offers particular advantages in patients with severe calcification of the ascending aorta [1]. However, randomized controlled trials to date have not demonstrated a clear superiority of off-pump over on-pump CABG. This controversy is partly fueled by concerns over methodological limitations in these trials, notably the inclusion of surgeons with relatively limited off-pump experience [2]. Moreover, one of the major advantages of OPCAB—the no-touch or anaortic technique—was not specifically evaluated in these trials. Later evidence has shown that no-touch aortic strategies can reduce the risk of postoperative stroke by nearly 60% compared to techniques using aortic side clamps [3,4].

There is broad consensus that the left internal mammary artery (LIMA) is the gold standard graft for CABG [1,5,6]. In multivessel disease, additional grafts are required, most commonly the saphenous vein, right internal mammary artery (RIMA), or radial artery (RA). For patients with high-grade coronary stenoses, current guidelines recommend complete arterial revascularization. The RA is believed to be particularly suitable for vessels with high-grade proximal stenosis, limited competitive flow, and for revascularization within the left coronary territory [1].

In our practice, the RA is the preferred second conduit because of its adequate length, durability, excellent long-term patency, and the advantage of simultaneous harvesting with the LIMA. In contrast, the RIMA is often too short to reach the right coronary artery branches, particularly in patients with dilated ventricles, and is more fragile. Furthermore, the use of bilateral internal mammary arteries has been associated with an increased risk of sternal wound complications, as reported in the ART trial [7].

Since its inception in 2004, our center has adopted the off-pump LIMA–RA Y-graft with a no-touch aortic strategy as the standard revascularization approach for all patients, irrespective of age, coronary anatomy, or the presence of left main disease—that is, for “all-comers.” The aim of this study is to present the short- and long-term outcomes of our 17-year experience with no-touch aorta OPCAB using the LIMA–RA Y-graft configuration.

## 2. Materials and Methods

This retrospective, single-center study included patients who underwent isolated off-pump coronary artery bypass grafting (OPCAB) with a no-touch aorta LIMA–radial artery (RA) Y-graft at Thorax Centrum Twente, Enschede, The Netherlands, between September 2004 and October 2021. The study adhered to the STROBE guidelines for observational research [8]. Patients who underwent surgery using cardiopulmonary bypass, underwent concomitant non-revascularisation related interventions, received venous or RIMA grafts, or those who had undergone previous cardiac surgeries were excluded from the analysis. Radial artery grafts were routinely harvested endoscopically [9].

The study was exempted from the Medical Research Involving Human Subjects Act by the Institutional Review Board of Medisch Spectrum Twente (K22-33), with a waiver for informed consent.

Baseline and procedural data were extracted from electronic patient records, and risk stratification was based on EuroSCORE I, EuroSCORE II, and Netherlands Heart Registration definitions [10]. Given the study’s “all-comer” design, selection bias was minimized, enhancing the generalizability of the findings. No formal sample size calculation was performed.

The primary endpoint was to assess short- and long-term survival, specifically 30-day and late survival rates. The secondary endpoint involved identifying hazard factors associated with postoperative 5-year survival, as well as conducting age-stratified survival analyses (categorized as <50 years, 50–59 years, 60–69 years, 70–79 years, and ≥80 years).

### 2.1. Surgical Technique

All procedures were performed via median sternotomy. The LIMA was harvested under direct vision using electrocoagulation, and the RA was harvested using an endoscopic technique. Distal anastomoses were completed with the aid of suction stabilization devices. Postoperatively, all patients received calcium-channel blockers, such as amlodipine, to prevent radial artery spasm, and single or dual antiplatelet therapy depending on the guidelines applicable at the time.

### 2.2. Statistical Analysis

Continuous variables were assessed for normal distribution using skewness/kurtosis measures and visual inspection of histograms. Normally distributed continuous variables are presented as means ± standard deviation, while non-normally distributed variables are reported as medians with interquartile ranges (25th–75th percentiles). Categorical variables were analyzed using the Fisher Exact test, and Chi-squared tests were employed where necessary due to hardware limitations. The Kruskal–Wallis test was applied to compare ordinal and non-parametric continuous variables across age groups, while one-way ANOVA was used for comparisons of normally distributed continuous variables.

Kaplan–Meier analysis and the log-rank test were used to evaluate survival times for the primary endpoint. For secondary analyses, hazard ratios (HR) were calculated using Cox regression models to determine factors associated with postoperative survival. Proportional hazards assumptions for the Cox regression model were assessed using Schoenfeld residuals. Age-stratified analyses were conducted for groups categorized by decades (<50, 50–59, 60–69, 70–79, and ≥80 years). Missing data were not imputed. Several important clinical variables had substantial proportions of missing data, including NYHA class (available in 848 of 2174 cases), EuroSCORE II (1153/2174), and preoperative serum creatinine level (800/2174).

Statistical significance was set at *p* < 0.05.

## 3. Results

**Figure 1:**
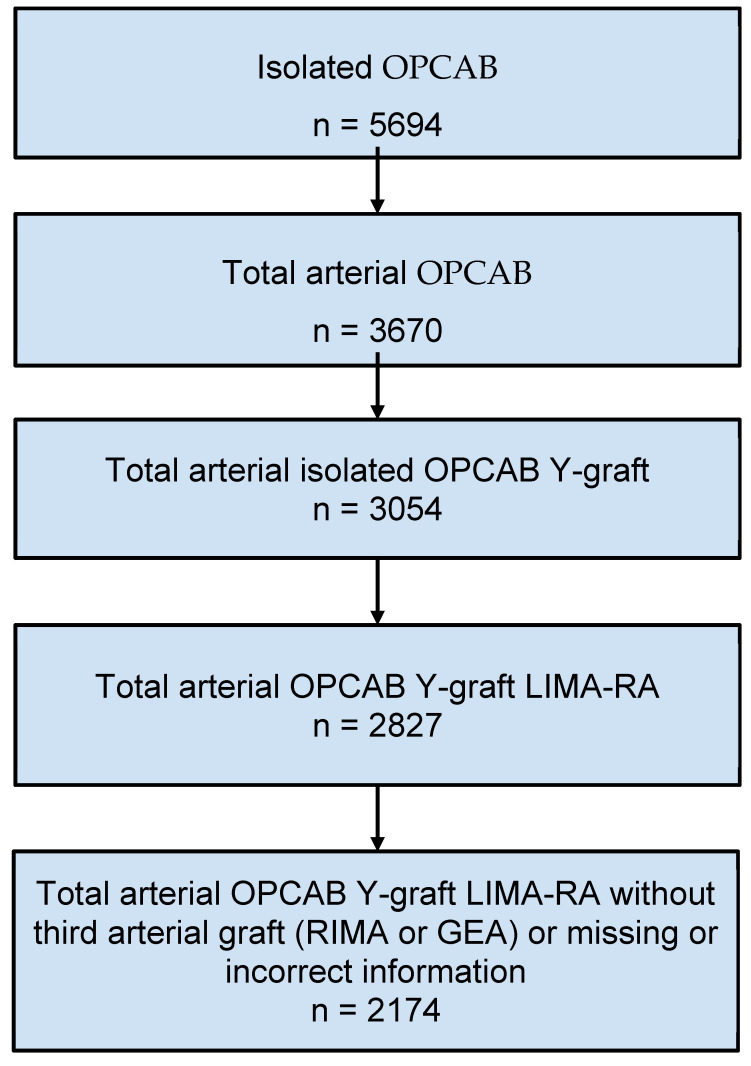
Patients who underwent off-pump coronary artery bypass surgery between September 2004 and October 2021 in Thorax Centrum Twente, Enschede, the Netherlands; OPCAB—off-pump coronary artery bypass grafting, LIMA—left internal mammary artery, RA—radial artery, RIMA—right internal mammary artery, GEA—gastroepiploic artery.

A total of 2174 patients underwent total arterial OPCAB with LIMA–RA Y-graft without any concomitant intervention (Figure 1). A total of 392 patients were excluded due to non-physiological values (e.g., implausible follow-up times or age due to entry errors) recorded in the database or lack of follow-up data. The median follow-up time for survival was 3266 days.

The mean age of the population was 65 ± 9.3 years, ranging from 36 to 87 years, with 85% of patients being male. Surgery was elective in 41% of patients, urgent in 58%, and emergent or salvage in 1%. All baseline characteristics are provided in Table 1 and Supplementary Table S1. In general, comorbidities increased with age. Survival follow-up was available for 99% of patients, with 13 patients lost to follow-up.

**Figure 2:**
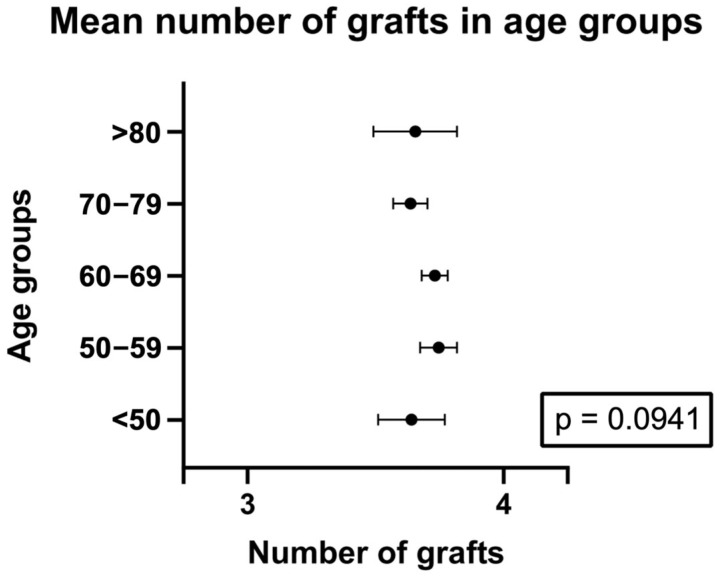
Graph presenting number of arterial anastomoses in age groups (mean and 95% confidence intervals) for no-touch aorta LIMA-RA total arterial OPCAB patients.

On average, 3.7 ± 0.79 distal arterial anastomoses were performed (Table 2, Figure 2). No differences in the number of anastomoses were observed between age groups. Notably, 58% of patients received four or more arterial anastomoses (Supplementary Table S3).

### 3.1. Survival

In-hospital mortality was 0.6%. Postoperative stroke without residual deficit occurred in 0.3% of patients and stroke with residual deficit in 0.2%, with no significant differences between age groups (Table 3, Supplementary Table S2).

**Figure 3:**
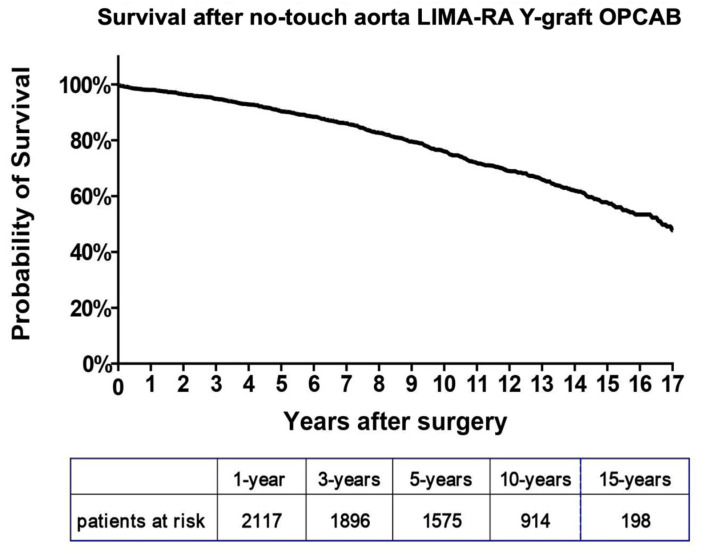
Graph presenting Kaplan–Meier survival probability after no-touch aorta LIMA-RA total arterial OPCAB patients between September 2004 and October 2021 in Thorax Centrum Twente, Enschede, the Netherlands.

The primary endpoint, 30-day survival, was 99%, and survival at five years was 90% (Figure 3, Supplementary Table S4).

### 3.2. Survival per Age Group

**Figure 4:**
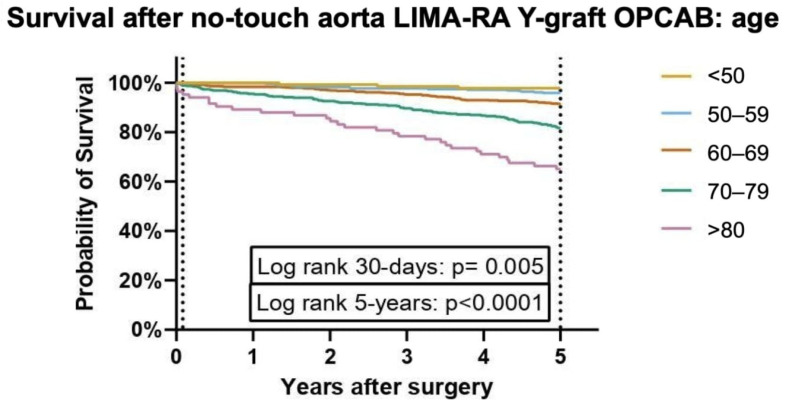
Graph presenting Kaplan–Meier survival probability after no-touch aorta LIMA-RA total arterial OPCAB patients categorised in age groups between September 2004 and October 2021 in Thorax Centrum Twente, Enschede, the Netherlands. Dotted lines indicate 30 days and 5 years, respectively.

A statistically significant difference in survival probability was observed between age groups, with older patients showing progressively lower survival curves (Figure 4). Survival probabilities by group are presented in Supplementary Table S4. Mortality rates increase progressively with age across multiple time intervals, with the highest rates consistently observed in the >80 age group. At 30 days, mortality is lowest in the <50 years group (0%) and gradually increases with age, reaching 3.4% in the >80 group (*p* = 0.005). By 1 year, the >80 group exhibits the highest mortality rate at 7.8% (*p* < 0.001). This trend continues, with the >80 age group demonstrating the highest mortality at each follow-up. At 3 years, 17.8% of individuals in the >80 group have died, compared to just 1.5% in the <50 group. At 5 years, 34.9% of the >80 group has died, compared to just 2.3% in the <50 group. The gap widens further at 10 years, where 84.1% of individuals over 80 have died, compared to only 10.7% in the <50 group. By 15 years, the >80 group has a 100% mortality rate, while the <50 group remains significantly lower at 42.9%. Detailed data on these mortality rates across the different age groups and time intervals are presented in Supplementary Table S3.

### 3.3. Mortality Predictors After No-Touch Aorta LIMA–RA Total Arterial OPCAB

**Figure 5:**
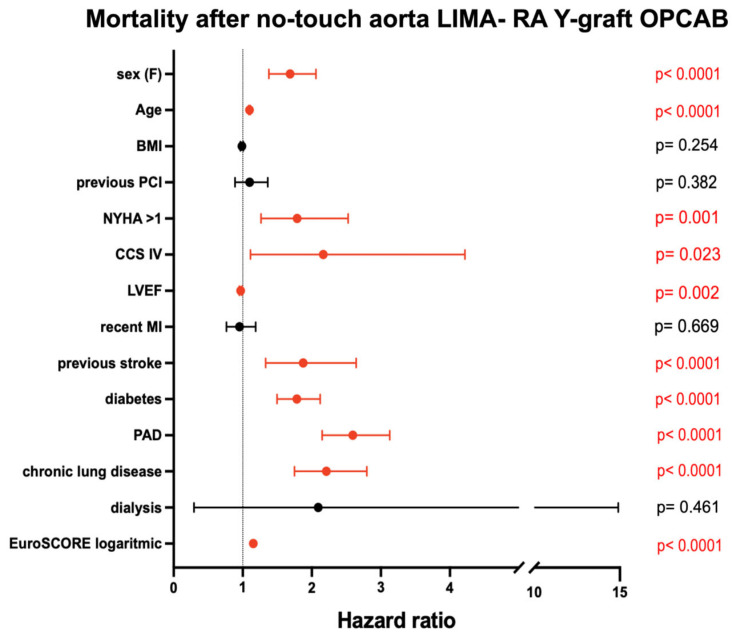
Cox regression hazard ratio after no-touch aorta LIMA-RA total arterial OPCAB. Proportional hazards assumptions for the Cox regression model were assessed using Schoenfeld residuals. The test did not reveal significant deviations from proportionality, supporting the validity of the model over the observed follow-up period.

Cox regression hazard ratio analysis identified several significant predictors of mortality after no-touch aorta LIMA–RA total arterial OPCAB (Figure 5). Female sex, older age, NYHA class > 1, CCS class IV, reduced left ventricular ejection fraction (LVEF), previous stroke, diabetes, peripheral artery disease (PAD), chronic lung disease, and higher EuroSCORE were all significantly associated with increased mortality (all *p* < 0.05). In contrast, body mass index (BMI), previous percutaneous coronary intervention (PCI), recent myocardial infarction (MI), and dialysis were not significantly associated with mortality.

## 4. Discussion

This study demonstrates that total arterial off-pump coronary artery bypass grafting (OPCAB) using the LIMA–RA Y-graft technique is a safe and effective revascularization strategy, offering excellent early and long-term outcomes across a broad patient population. In a large, single-center cohort of 2174 patients, we observed remarkably low in-hospital mortality (0.6%) and a minimal incidence of postoperative stroke, with consistent results across age groups. Thirty-day survival reached 99%, and five-year survival was 90%, confirming the durability and safety of this approach. These findings reinforce the benefits of total arterial grafting and suggest that, when performed in experienced hands, OPCAB can be applied widely without compromising outcomes, even in elderly or high-risk patients. The short- and mid-term mortality in our cohort was better than those reported in the landmark ART randomized controlled trial [7], 0.6 vs. 1.2% at 30 days and 10% vs. 12.2–12.7% at 5 years.

We deliberately excluded patients who received a third arterial graft (e.g., RIMA or GEA) or a venous graft in order to minimize the confounding influence of known additional morbidities associated with these conduits, such as mediastinitis with bilateral internal mammary artery use, abdominal complications with GEA, or wound issues with saphenous vein grafts. Instead, we focused the analysis on outcomes associated with our preferred strategy: the LIMA–RA Y-graft configuration.

However, as expected, there was a clear age-dependent trend in long-term survival. Patients in older age groups had progressively lower survival rates at each follow-up interval. While the <50 years group had a near-zero mortality rate at 30 days, the >80 years group experienced a significantly higher mortality rate, starting at 3.4% at 30 days and escalating to 100% by 15 years. These findings are consistent with previous studies demonstrating that advancing age is one of the most robust predictors of mortality after coronary artery bypass grafting (CABG) [11,12]. However, it seems obvious that long-term survival in octogenarians is expected to be low and not necessarily attributed to coronary artery disease. These patients still benefit from low short-term mortality and a low risk of a devastating complication of postoperative stroke.

Age-related mortality differences observed in this study align with the results from the Cox regression hazard ratio analysis, which identified older age, female sex, poor functional status (NYHA class > 1, CCS class IV), comorbidities such as diabetes and peripheral artery disease (PAD), and a reduced left ventricular ejection fraction (LVEF) as significant predictors of mortality. These factors have been widely recognized as risk factors for poor long-term outcomes in CABG patients and emphasize the importance of a comprehensive preoperative assessment to identify high-risk individuals [13,14]. Interestingly, factors such as body mass index (BMI), previous percutaneous coronary intervention (PCI), recent myocardial infarction (MI), and dialysis were not found to be significantly associated with mortality in this cohort, which may suggest that the benefits of total arterial grafting are not attenuated by these conditions.

In terms of surgical technique, the use of total arterial grafting, specifically the LIMA–RA Y-graft, remains associated with favorable long-term outcomes, even in older patients. The LIMA graft, in particular, is known for its long-term patency and is considered the gold standard in coronary artery bypass surgery [1].

In experienced centers, off-pump coronary artery bypass (OPCAB) can be safely performed in all patients regardless of age or left ventricular function. The key is surgical expertise and performing OPCAB routinely in all patients. We occasionally use an intraaortic balloon pump or extracorporeal circulation (ECC-assisted CABG) in hemodynamically very unstable patients.

Importantly, this approach allows for extensive revascularization. In our cohort, the mean number of distal anastomoses was 3.7 per patient (58% of patients receiving four or more arterial anastomoses), demonstrating that no-touch total arterial OPCAB does not have to compromise the “completeness” of the revascularisation, especially in experienced hands.

These findings support the use of no-touch aorta, total arterial OPCAB as a universally effective revascularization strategy. Particularly in older patients, the technique offers excellent short-term outcomes with remarkably low perioperative stroke rates—a critical factor in this high-risk population. The approach demonstrates safety and efficacy across all age groups, making it an attractive default strategy rather than one limited to selected cases.

This study has several limitations inherent to its retrospective and single-center design. Although the large cohort and standardized surgical technique strengthen the findings, the absence of a control group (e.g., on-pump or hybrid CABG patients) limits direct comparisons. As such, it does not allow for definitive conclusions regarding the superiority or equivalence of the no-touch aorta LIMA–RA Y-graft OPCAB technique relative to on-pump CABG or alternative revascularization strategies. While this limits comparative inference, the strength of the study lies in its large, unselected patient cohort and consistent application of a standardized surgical technique over a 17-year period. The findings should be interpreted as real-world outcome data from a high-volume center with extensive experience in total arterial OPCAB. These results must be interpreted with caution and may not be generalizable or reproducible in centers with limited experience in this surgical approach. Furthermore, data on certain postoperative variables, such as neurocognitive outcomes and long-term quality of life, were not systematically collected. The all-comers strategy improves generalizability, but selection bias cannot be entirely excluded due to the exclusion of patients with missing graft data. Finally, while long-term survival was robustly analyzed, cause-specific mortality was not available for most patients, which limits interpretation of graft-related vs. non-cardiac death over time. It may have had an impact on the results, especially in the elderly, frail, and multi-morbid patients. Moreover, the data on the history of smoking, dyslipidemia, or hypertension were not reliably reported and thus not presented and analyzed in this study.

In conclusion, no-touch aorta, total arterial OPCAB using the LIMA–RA Y-graft performed in a high-volume off-pump coronary artery bypass grafting center is a safe and effective revascularization strategy for a broad spectrum of patients, including those with advanced age and comorbidities. However, we underline the importance of establishing a structured OPCAB program to perform it safely in all patient groups.

## Figures and Tables

**Table 1:** Preoperative demographics for the overall group and per age category.

	Overall n = 2174	<50 Years n = 142	50–59 Years n = 466	60–69 Years n = 856	70–79 Years n = 594	>80 Years n = 116	*p*-Value
Age, years	65 ± 9.3	45 ± 2.9	55 ± 2.7	64 ± 2.7	74 ± 2.6	82 ± 2	<0.001
Sex, male, n (%)	1844 (85)	128 (90)	434 (93)	731 (85)	467 (79)	84 (72)	<0.001
BMI, kg/m^2^	28 ± 4.1	28 ± 4.2	29 ± 4.1	28 ± 3.9	28 ± 4.3	27 ± 3.5	<0.001
NYHA, n (%) *							0.026
1	483 (57)	18 (64)	80 (53)	171 (59)	186 (59)	28 (42)	
2	52 (30)	8 (29)	45 (30)	82 (29)	93 (30)	24 (36)	
3	106 (13)	2 (7.1)	24 (16)	34 (12)	32 (10)	14 (21)	
4	7 (0.8)	0	1 (0.7)	1 (0.3)	4 (1.3)	1 (1.5)	
Euroscore I, log	2.2 [1.3–3.7]	1.2 [0.9–1.5]	1.3 [0.9–1.7]	1.8 [1.4–2.8]	3.6 [2.5–6.05]	7.3 [5.5–11]	<0.001
Euroscore II *	1 [0.7–1.5]	0.7 [0.6–0.8]	0.8 [0.6–1.1]	0.9 [0.7–1.2]	1.3 [0.9–1.8]	2 [1.3–3.3]	<0.001
LVEF, %	55 [40–55]	55 [43–55]	55 [40–55]	55 [50–55]	55 [40–55]	55 [40–55]	0.345
Diabetes, n (%)	551 (26)	30 (21.6)	105 (23)	218 (26)	171 (29)	27 (24)	
Peripheral artery disease, n (%)	333 (15)	12 (8.5)	46 (9.9)	127 (15)	118 (20)	30 (26)	<0.001
Recent MI, n (%)	448 (21)	35 (25)	113 (24)	161 (19)	108 (18)	31 (27)	0.020
Previous stroke, n (%)	91 (4.2)	3 (2.1)	13 (2.8)	32 (3.7)	37 (6.2)	6 (5.2)	0.037
Previous PCI, n (%)	397 (18)	28 (20)	85 (18)	158 (19)	107 (18)	19 (16)	0.972
Chronic lung disease, n (%)	186 (8.6)	8 (5.6)	26 (5.6)	78 (9.1)	63 (11)	11 (9.5)	0.028
CCS Class IV, n (%)	51 (2.3)	0	11 (2.4)	15 (1.8)	17 (2.9)	8 (6.9)	0.006
Dialysis, n (%)	2 (0.1)	1 (0.7)	0	1 (0.1)	0	0	0.269
Urgency, n							0.007
Elective, n (%)	305 (41)	10 (59)	50 (38)	88 (36)	138 (48)	19 (32)	
Urgent, n (%)	427 (58)	7 (41)	79 (60)	155 (63)	146 (51)	40 (67)	
Emergent, n (%)	7 (0.9)	0	2 (1.5)	2 (0.8)	2 (0.7)	1 (1.7)	
Salvage, n (%)	1 (0.1)	0	1 (0.8)	0	0	0	

* Recorded from March 2015. Normally distributed continuous variables were noted as mean ± standard deviation. Non-parametric variables were noted as median [25th percentile–75th percentile]. BMI = body mass index; CCS = Canadian Cardiovascular Society grading of angina pectoris; DM = diabetes mellitus; LVEF = left ventricular ejection fraction; MI = myocardial infarction; NYHA = New York Heart Association scale; PAD = peripheral artery disease; PCI = percutaneous coronary intervention.

**Table 2:** Mean number of grafts overall and per age category.

	Overall n = 2174	<50 Years n = 142	50–59 n = 466	60–69 n = 856	70–79 n = 594	>80 n = 116
Mean number of grafts	3.7 ± 0.8	3.6 ± 0.8	3.8 ± 0.8	3.7 ± 0.8	3.6 ± 0.8	3.7 ± 0.9

**Table 3:** Postoperative data for the overall group and per age category.

	Overall n = 2174	<50 Years n = 142	50–59 n = 466	60–69 n = 856	70–79 n = 594	>80 n = 116	*p*-Value
Death in hospital, n (%)	13 (0.6)	0	0	4 (0.5)	5 (0.8)	4 (3.4)	0.005
Perioperative MI, n (%)							0.130
Enzymatic MI, n (%)	18 (0.8)	1 (0.7)	6 (1.3)	4 (0.5)	5 (0.9)	2 (1.8)	
Transmural MI, n (%)	12 (0.6)	1 (0.7)	0	4 (0.5)	7 (1.2)	0	
Arm wound infection during admission, n (%)	6 (0.3)	1 (0.7)	0	1 (0.1)	3 (0.5)	1 (0.9)	0.102
Pulmonary infection, n (%)	117 (5.4)	7 (4.9)	18 (3.9)	49 (5.7)	33 (5.6)	10 (8.6)	0.297
Urinary tract infection, n (%)	16 (0.7)	0	0	7 (0.8)	8 (1.3)	1 (0.9)	0.070
Respiratory insufficiency, n (%)	38 (1.7)	0	2 (0, 4)	16 (1.9)	18 (3)	2 (1.7)	0.007
CVA with residual trauma, n (%)	5 (0.2)	1 (0.7)	0	2 (0.2)	1 (0.2)	1 (0.9)	0.188
CVA without residual trauma, n (%)	6 (0.3)	0	0	2 (0.2)	3 (0.5)	1 (0.9)	0.275
Kidney failure, n (%)	5 (0.2)	1 (0.7)	0	3 (0.4)	1 (0.2)	0	0.444
Rhythm problems, n (%)	503 (23)	8 (5.6)	53 (11)	205 (24)	191 (32)	46 (40)	<0.001
Rethoracotomy within 30 days, n (%)	21 (0.9)	0	1 (0.2)	8 (1)	9 (1.5)	3(2.6)	0.094
Bleeding tamponade, n (%)	14 (0.6)	0	1 (0.2)	4 (0.5)	7 (1.2)	2 (1.7)	
Cardiac problems—surgery with and without ECC, n (%)	6 (0.3)	0	0	4 (0.5)	2 (0.3)	0	
Other cause, n (%)	1 (0)	0	0	0	0	1 (0.9)	
Deep sternal wound infection, n (%)	5 (0.2)	0	1 (0.7)	2 (0.8)	2 (0.7)	0	1.000

CVA = Cerebrovascular accident.

## Data Availability

Data supporting reported results can be obtained from the corresponding author.

## Supplementary Materials

The following supporting information can be downloaded at: https://www.mdpi.com/article/10.3390/jcm14144878/s1, Table S1: Preoperative demographics for the overall group and per age category; Table S2: Postoperative data for the overall group and per age category; Table S3: Number of arterial anastomosis overall and per age category; Table S4: Mortality after specific time.
