# Supplementary material for: No-Touch Aorta Off-Pump LIMA-Radial Artery Y-Graft CABG as a Safe Strategy for All-Comers: Long-Term Survival
Source: J Clin Med. 2025 Jul 9;14(14):4878. doi: 10.3390/jcm14144878 (PMC12295065; doi:10.3390/jcm14144878)
Supplement: Supplementary file 1 [file jcm-14-04878-s001.zip › jcm-3737738-supplementary.pdf]

Table S1. Preoperative demographics for the overall group and per age category

	Overall n = 2174	< 50 years n=142	50-59 years n=466	60-69 years n=856	70-79 years n=594	> 80 years n=116	p-value
Age, years	65 ± 9.3	45 ± 2.9	55 ± 2.7	64 ± 2.7	74 ± 2.6	82 ± 2	<0.001
Sex, male, n (%)	1844 (85)	128 (90)	434 (93)	731 (85)	467 (79)	84 (72)	<0.001
BMI, kg/m2	28 ± 4.1 n=2066	28 ± 4.2 n=133	29 ± 4.1 n=433	28 ± 3.9 n=825	28 ± 4.3 n=564	27 ± 3.5 n=111	<0.001
NYHA, n (%) *							0.026
1	483 (57)	18 (64.3)	80 (53.3)	171 (59.4)	186 (59)	28 (41.8)	
2	52 (29.7)	8 (28.6)	45 (30)	82 (28.5)	93 (29.5)	24 (35.8)	
3	106 (12.5)	2 (7.1)	24 (16)	34 (11.8)	32 (10.2)	14 (20.9)	
4	7 (0.8)	0	1 (0.7)	1 (0.3)	4 (1.3)	1 (1.5)	
Euroscore_log	2.2 [1.33 - 3.73] n= 2164	1.22 [0.88 - 1.51]	1.3 [0.88 - 1.68] n=463	1.84 [1.4 - 2.84] n=854	3.57 [2.52 - 6.05] n=589	7.25 [5.45 - 10.91]	<0.001
Euroscore II *	0.98 [0.72 - 1.49] n=1153	0.67 [0.55- 0.79] n= 55	0.76 [0.55 - 1.06] n=232	0.88 [0.69 - 1.22] n=411	1.25 [0.92 - 1.8] n=373	1.97 [1.31 - 3.25] n=82	<0.001
LVEF, %	55 [40 - 55] n=745	55 [43 - 55] n=16	55 [40 - 55] n=134	55 [50 - 55] n=250	55 [40.75 - 55] n=286	55 [40 - 55] n=59	0.345
DM, n (%)	551 (25.7) n=2145	30 (21.6) n=139	105 (23) n=456	218 (25.7) n=848	171 (29.1) n=587	27 (23.5) n=115	
PAD, n (%)	333 (15.3)	12 (8.5)	46 (9.9)	127 (14.8)	118 (19.9)	30 (25.9)	<0.001
recent MI, n (%)	448 (20.6)	35 (24.6)	113 (24.2)	161 (18.8)	108 (18.2)	31 (26.7)	0.020
Previous stroke, n (%)	91 (4.2)	3 (2.1)	13 (2.8)	32 (3.7)	37 (6.2)	6 (5.2)	0.037
Previous PCI, n (%)	397 (18.3)	28 (19.7)	85 (18.2)	158 (18.5) n=855	107 (18)	19 (16.4)	0.972
Chronic lung disease, n (%)	186 (8.6)	8 (5.6)	26 (5.6)	78 (9.1)	63 (10.6)	11 (9.5)	0.028
CCS IV, n (%)	51 (2.3)	0	11 (2.4)	15 (1.8)	17 (2.9)	8 (6.9)	0.006
Dialysis, n (%)	2 (0.1)	1 (0.7)	0	1 (0.1)	0	0	0.269
Urgency, n	n=740	n=17	n=132	n=245	n=286	n=60	0.007
Elective, n (%)	305 (41.2)	10 (58.8)	50 (37.9)	88 (35.9)	138 (48.3)	19 (31.7)	
Urgent, n (%)	427 (57.7)	7 (41.2)	79 (59,8)	155 (63.3)	146 (51)	40 (66.7)	
Emergent, n (%)	7 (0.9)	0	2 (1.5)	2 (0.8)	2 (0.7)	1 (1.7)	

Salvage, n (%)	1 (0.1)	0	1 (0.8)	0	0	0

* Recorded from March 2015
Normally distributed continuous variables were noted as mean ± standard deviation. Non-parametric variables were noted as median [25th percentile - 75th percentile].
BMI = Body mass index; CCS = Canadian Cardiovascular Society grading of angina pectoris; DM = Diabetes -mellitus; LVEF = Left ventricular ejection fraction; MI = Myocardial infarction; NYHA = New York Heart Association scale; PAD = Peripheral artery disease; PCI = Percutaneous coronary intervention.

Table S2. Postoperative data for the overall group and per age category.

	Overall n = 2174	< 50 years n=142	50-59 n=466	60-69 n=856	70-79 n=594	> 80 n=116	p-value
Death in hospital, n (%)	13 (0.6)	0	0	4 (0.5)	5 (0.8)	4 (3.4)	0.005
Perioperative MI, n (%)	n=2131	n=16	n=129	n=247	n=284	n=59	0.130
Enzymatic MI, n (%)	18 (0.8)	1 (0.7)	6 (1.3)	4 (0.5)	5 (0.9)	2 (1.8)	
Transmural MI, n (%)	12 (0.6)	1 (0.7)	0	4 (0.5)	7 (1.2)	0	
Arm wound infection during admission, n (%)	6 (0.3)	1 (0.7)	0	1 (0.1)	3 (0.5)	1 (0.9)	0.102
Pulmonary infection, n (%)	117 (5.4)	7 (4.9)	18 (3.9)	49 (5.7)	33 (5.6)	10 (8.6)	0.297
Urinary tract infection, n (%)	16 (0.7)	0	0	7 (0.8)	8 (1.3)	1 (0.9)	0.070
Respiratory insufficiency, n (%)	38 (1.7)	0	2 (0.4)	16 (1.9)	18 (3)	2 (1.7)	0.007
CVA with residual trauma, n (%)	5 (0.2)	1 (0.7)	0	2 (0.2)	1 (0.2)	1 (0.9)	0.188
CVA without residual trauma, n (%)	6 (0.3)	0	0	2 (0.2)	3 (0.5)	1 (0.9)	0.275
Kidney failure, n (%)	5 (0.2)	1 (0.7)	0	3 (0.4)	1 (0.2)	0	0.444
Rhythm issue, n (%)	503 (23.1)	8 (5.6)	53 (11.4)	205 (23.9)	191 (32.2)	46 (39.7)	<0.001
Rethoracotomy within 30 days, n (%)	21 (0.9)	0	1 (0.2)	8 (1)	9 (1.5)	3(2.6)	0.094
Bleeding tamponade, n (%)	14 (0.6)	0	1 (0.2)	4 (0.5)	7 (1.2)	2 (1.7)	
Cardiac problems- surgery with or without ECC, n (%)	6 (0.3)	0	0	4 (0.5)	2 (0.3)	0	
Other cause, n (%)	1 (0)	0	0	0	0	1 (0.9)	
Deep sternal wound infection, n (%)	5 (0.2) n=763	0 n=22	1 (0.7) n=137	2 (0.8) n=252	2 (0.7) n=292	0 n=60	1.000

CVA = Cerebrovascular accident

Table S3. Number of arterial anastomosis overall and per age category.

	Overall n = 2174	< 50 years n=142	50-59 n=466	60-69 n=856	70-79 n=594	> 80 n=116	p-value
arterial anastomosis, n (%)							
2	81 (3.7)	8 (5.6)	15 (3.2)	20 (2.3)	28 (4.7)	10 (8.6)	
3	834 (38.4)	53 (37.3)	166 (35.6)	322 (37.6)	253 (42.6)	40 (34.5)	
4	948 (43.6)	64 (45.1)	214 (45.9)	391 (45.7)	231 (38.9)	48 (41.4)	
5	284 (13.1)	16 (11.3)	65 (13.9)	114 (13.3)	73 (12.3)	16 (13.8)	
6	24 (1.1)	1 (0.7)	5 (1.1)	9 (1.1)	7 (1.2)	2 (1.7)	
7	3 (0.1)	0	1 (0.2)	0	2 (0.3)	0	

Table S4. Mortality after specific time.

	Overall n = 2174	< 50 years n=142	50-59 n=466	60-69 n=856	70-79 n=594	> 80 n=116	p-value
MORTALITY							
30- day, n (%)	12 (0.6) n=2160	0 n=139	1 (0.2) n= 461	2 (0.2) n=852	5 (0.8) n=592	4 (3.4)	0.005
1 year, n (%)	42 (1.9) n=2160	0 n=139	1 (0.2) n=461	12 (1.4) n=852	20 (3.4) n=592	9 (7.8)	<0.001
3 years, n (%)	110 (5.5) n=2007	2 (1.5) n=137	9 (2.1) n=433	35 (4.3) n= 807	46 (8.7) n=529	18 (17.8) n=101	<0.001
5 years, n (%)	192 (10.9) n= 1767	3 (2.3) n=133	16 (4.1) n=386	63 (8.7) n=722	81 (18.3) n=443	29 (34.9) n=83	<0.001
10 years, n (%)	405 (30.7) n= 1319	11 (10.7) n= 103	41 (14.3) n=286	137 (24.9) n=550	163 (51.4) n=317	53 (84.1) n=63	<0.001
15 years, n (%)	544 (73.3) n= 742	15 (42.9) n=35	57 (46) n=124	188 (67.9) n=277	225 (91.9) n=247	59 (100) n=59	<0.001
